# Complete mitochondrial genome of the hybrid loach of *Paramisgurnus dabryanus* ssp. (female) and *Misgurnus anguillicaudatus* (male)

**DOI:** 10.1080/23802359.2018.1450654

**Published:** 2018-03-13

**Authors:** Xia Liang, Daoyu Zhu, Xiaodong Li, Kejun Cai, Haili Zhang, Guosong Zhang

**Affiliations:** a Key Laboratory for Physiology Biochemistry and Application, School of Agriculture and Bioengineering, Heze University, Heze, Shandong, China;; b School of Chemistry and Bioengineering, Hechi University, Yizhou, Guangxi, China;; c Department of Life Science, Huzhou University, Huzhou, Zhejiang, China

**Keywords:** *Paramisgurnus*, *Misgurnus anguillicaudatus*, Taiwanese loach, hybrid loach

## Abstract

The hybrid loach of *P. dabryanus* ssp. (female) and *M. anguillicaudatus* (male) has the desirable trait of growth performance. There is no report of the complete genome of this hybrid. In this study, the complete mitochondrial genome of this hybrid loach was obtained, and the genome is 16,569 bp in length, including two ribosomal RNA genes. Thirteen proteins-coding genes, 22 transfer RNA genes, and a non-coding control region, the gene composition and order of which was similar to most reported from other vertebrates. Sequence analysis showed that the overall base composition is 29.5% for A, 27.5% for T, 26.4% for C, and 16.6% for G. The sequence is a slight A + T bias of 57.0%. The phylogenetic tree showed that the hybrid loach to be one of *Paramisgurnus*, and the relationships of *M. anguillicaudatus* were closer. Also the mitochondrial genome sequence of loach were aligned by BLAST, compared with Cobitinae the sequence similarity could reach >90%, and the similarity to *Paramisgurnus* was >99%. Mitogenome information from this study could be a useful basis for conservation and phylogenetics of this hybrid loach.

Taiwanese loach (*Paramisgurnus dabryanus* ssp.) was bred in Formosa and then widely cultivated in China. The Taiwanese loach and *Misgurnus anguillicaudatus* is a commercially important Cypriniformes fish species in Asia. The hybrid loach of *P. dabryanus* ssp. (female) and *M. anguillicaudatus* (male) has the desirable trait of growth performance. Recently, farming scale of the hybrids has been gradually increased in Asia, suggesting a promise of a new variety for loach. There is no report of the complete genome of this hybrid. Therefore, it is very important to characterize the complete mitogenome of this species, which can be utilized in research on taxonomic resolution, population genetic structure and phylogeography, and phylogenetic relationship (Zhang et al. 2017).

In this study, we sequenced the complete mitogenome of this hybrid with a GenBank accession number MG735453. The voucher specimen was collected from Zhili Fanyi aquaculture base, north latitude 30°22″ and east longitude 120°25″, Huzhou city, China, which were stored in biology herbarium of Heze University. Its tailfins were preserved in 95% alcohol. All DNA were extracted using phenol–chloroform extraction methods and stored at –80 °C (Chen and Wang 2015). The complete mitochondrial genome sequences were amplified by primers which were initially published *P. dabryanus* (Dai et al. 2016).

The complete mitochondrial genome was 16,569 bp in length, including two ribosomal RNA genes, 13 proteins-coding genes, 22 transfer RNA genes, and a non-coding control region, which was similar to other fishes. The overall base composition was 29.5% for A, 27.5% for T, 26.4% for C, and 16.6% for G. The sequence was a slight A + T bias of 57.0%. Most of the protein genes used ATG as the initiation condons (ND1, ND2, COX2, ATP8, ATP6, COX3, ND3, ND4L, ND4, ND5, and Cytb), except for COX1 and ND6 genes, which used GTG and ATA instead of ATG. Eight protein-coding genes ended with complete termination codons, TAA (ND1, ND2, COX1, ATP8, ATP6, ND3, ND4L, and ND5), gene ND4 used TAG as termination codons, and ND6 use CAT instead of TAA. COX2, COX3, and Cytb shares the incomplete stop codons T. Except for eight tRNA (tRNA^Ser^, tRNA^Pro^, tRNA^Glu^, tRNA^Tyr^, tRNA^Cys^, tRNA^Asn^, tRNA^Ala^, and tRNA^Gln^) and the ND6 genes encoded on the L-strand, the other genes were encoded on the H-strand. The 21 kinds of tRNA, tRNA^Leu^, and tRNA^Ser^ repeated in the complete mitochondrial genome. This feature is similar to other fish mitochondrial genes. The complete mitogenome sequence had 16s RNA (1678 bp) and 12s RNA (953 bp), which were located between tRNA^Phe^ with tRNA^Leu(UUR)^ and were separated by tRNA^Val^ genes. The location is same with most vertebrates that have high conservative. As in most vertebrates, two non-coding regions were found in the hybrid loach mitogenome, the only CR (913 bp) gene located between tRNA^Pro^ and tRNA^Phe^, and an OL (30 bp) was located between tRNA^Asn^ and tRNA^Cys^. The 21 tRNA genes, ranging from 66 to 76 bp in size, except for tRNA^Ser(AGY)^, which lacks a dihydrouridine arm, could be folded into cloverleaf secondary structure. The origin of L-strand was in the WANCY region including five tRNA genes (tRNA^Trp^, tRNA^Ala^, tRNA^Asn^, tRNA^Cys^, and tRNA^Tyr^) and it can fold into a stem-loop secondary structure with the conserved motif 50-GCCGG-30.

To determine taxonomic status of the hybrid loach, we performed the phylogenetic relationship of this loach stock with other natural populations in loach as inferred by entire mitogenome (Sang et al. 2017). The phylogenetic tree showed that the hybrid loach to be one of *Paramisgurnus*, and the relationships of *M. anguillicaudatus* were closer (Figure 1). Also the mitochondrial genome sequence of loach were aligned by BLAST, compared with Cobitinae the sequence similarity could reach >90%, and the similarity to *Paramisgurnus* was >99%.

**Figure 1.**
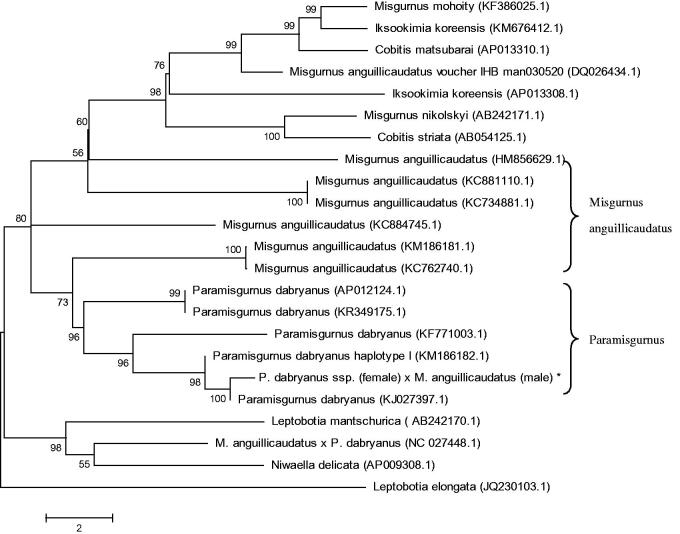
Phylogenetic relationship of the hybrid loach of *P. dabryanus ssp.* (female) and *M. anguillicaudatus* (male) stock with other loach as inferred by entire mitogenome. *The hybrid loach (accession number: MG735453) in the position of the evolutionary tree. Trees were reconstructed using MEGA 7 program (ver. 7.0.26) with neighbour-joining method. Numbers above branches are bootstrap values by 1000 replicates. The phylogenetic tree showed that the hybrid loach to be one of *Paramisgurnus*, and the relationships of *M. anguillicaudatus* were closer.
